# *In vivo* high-resolution fluorescence microendoscopy for ovarian cancer detection and treatment monitoring

**DOI:** 10.1038/sj.bjc.6605436

**Published:** 2009-11-17

**Authors:** W Zhong, J P Celli, I Rizvi, Z Mai, B Q Spring, S H Yun, T Hasan

**Affiliations:** 1 Wellman Center for Photomedicine, Massachusetts General Hospital, Harvard Medical School, Boston, MA 02114, USA; 2 Thayer School of Engineering, Dartmouth College, Hanover, NH 03755, USA

**Keywords:** imaging, optical fibre, photodynamic therapy (PDT), benzoporphyrin-derivative monoacid ring A (BPD-MA)

## Abstract

**Background:**

In patients with advanced ovarian cancer (OvCa), microscopic residual tumour nodules that remain after surgical debulking frequently escape detection by current treatment assessment methods and lead to disease recurrence. The aim of this study was to evaluate the use of high-resolution fibre-optic fluorescence imaging of the clinically approved photodynamic therapy (PDT) agent benzoporphyin-derivative monoacid ring A (BPD-MA) for detection of microscopic OvCa and for monitoring treatment response.

**Methods:**

Our fluorescence microendoscope consists of a flexible imaging fibre coupled to a custom epi-fluorescence system optimised for imaging BPD-MA, which, after a single administration, serves as both an imaging agent and a light-activated therapeutic agent. After characterisation in an *in vitro* OvCa 3D model, we used the flexible imaging fibre to minimally invasively image the peritoneal cavity of a disseminated OvCa murine model using BPD-MA administered intraperitoneally (i.p.). To evaluate longitudinal changes in response to treatment, we compared sets of images obtained before and after PDT with those from untreated mice imaged at the same time points.

**Results:**

By comparison with histopathology, we report an 86% sensitivity for tumour detection *in vivo* using the microendoscope. Using a custom routine to batch process-image data in the monitoring study, treated mice exhibited an average decrease of 58.8% in tumour volumes compared with an increase of 59.3% in untreated controls (*P*<0.05).

**Conclusions:**

Our findings indicate the potential of this approach as a reporter of treatment outcome that could aid in the rational design of strategies to mitigate recurrent OvCa.

In this study, we demonstrate the capability of a custom-built fluorescence microendoscope to sensitively detect microscopic ovarian cancer (OvCa) tumour nodules and monitor treatment response *in vivo.* This effort is motivated by a vital clinical need for improved reassessment imaging for this disease. The dismal survival rates for OvCa are largely because of the high proportion of patients diagnosed at a late stage, which is characterised by disseminated nodular studding throughout the peritoneal cavity and presents challenges for treatment (Jemal *et al*, 2008; Cho and Shih, 2009). Despite initial responses to standard tumour debulking and chemotherapy, many of these patients continue to harbour occult disseminated disease without immediate clinical evidence of tumour recurrence because of the microscopic size of residual nodules (Berek and Bast, 2003). Second-look laparotomy allows the surgeon to check for persistent residual tumours, although even this highly invasive surgical reassessment suffers from a false-negative rate of up to 50% (Selman and Copeland, 1999; DiSaia and Creasman, 2002). These laparotomies are typically performed 6 months to 2 years after treatment, at which point, persistent disease, if present, has progressed to the point that is readily evident to the surgeon and the possibility of an earlier intervention has been precluded. Non-invasive diagnostic techniques, such as CA125 levels, ultrasound, computerised tomography, magnetic resonance imaging (MRI), and positron emission tomography (PET), which could in principle provide the benefit of more timely feedback, have all been shown to be less sensitive than reassessment surgeries (Pectasides *et al*, 1991; Sugiyama *et al*, 1996; Selman and Copeland, 1999; Morrow, 2000; Rose *et al*, 2001; Tammela and Lele, 2004). Hence, there is a vital un-met need for a new minimally invasive imaging approach with sufficient sensitivity and resolution to detect sub-millimetre OvCa nodules early in the treatment cycle, thus providing the basis for more timely interventions to mitigate recurrent disease.

To address the above requirements, several key features of optical imaging warrant consideration, including its inherently high resolution and the ability to achieve greater specificity by use of fluorescent contrast agents (Prasad, 2003; Vo-Dinh, 2003). Furthermore, technological advances in fibre optic fluorescence imaging, a modality that allows investigators to reach into body cavities through minimally invasive endoscopes, have considerably broadened the applications of *in vivo* optical imaging (Gmitro and Aziz, 1993; MacAulay *et al*, 2004; Flusberg *et al*, 2005). Although second-look laparoscopy by white light imaging has received significant attention as a minimally invasive procedure to detect OvCa recurrence (Davila and Estape, 2001), it suffers from a high false-negative rate of 55% (Ozols *et al*, 1981) and also fails to provide the requisite optical resolution and sensitivity to detect the smallest residual tumours.

An approach to OvCa detection that has shown promise is the use of photosensitisers (PS), traditionally used for photodynamic therapy (PDT), to generate tumour-selective fluorescence contrast. Although fluorescence imaging of non-therapeutic imaging agents has also shown impressive potential for OvCa detection (Sheth *et al*, 2009), PS imaging offers a unique advantage. Upon wavelength-specific activation of the PS, fluorescence emission for diagnostic imaging and cytotoxic species (through intersystem crossing to the triplet state) for tissue destruction can both be produced. Previous studies that used PS (ALA-induced protoporphyrin IX and/or hexaminolaevulinate) for OvCa imaging were successful in detecting significantly more lesions than white light imaging (Chan *et al*, 2002; Ludicke *et al*, 2003; Loning *et al*, 2004), with a ratio of fluorescence intensity of neoplastic to normal tissues of ∼4 (Chan *et al*, 2002; Ludicke *et al*, 2003). The average size of the optically biopsied metastatic lesions was 1.0 mm (range: 0.3–2.5) compared with 1.5 mm (range: 0.5–2.9) with white light illumination (Chan *et al*, 2002). However, in the absence of instrumentation for high-resolution *in vivo* microscopy in these studies, the smallest occult nodules (<300 *μ*m) were not within the reported size range.

It is our thesis that if the demonstrated capability of PS fluorescence for detection of OvCa nodules is combined with high-resolution fibre-optic microscopy, it would allow for highly sensitive and minimally invasive detection of microscopic disease. To this end, we developed a custom fluorescence microendoscope (Figure 1) optimised for imaging the weak fluorescence emission from benzoporphyin-derivative monoacid ring A (BPD-MA) (delivered in its liposomal formulation known as verteporfin or Visudyne). BPD-MA is clinically approved as a therapeutic PDT agent and is used in our study to provide both diagnostic and therapeutic capabilities after a single administration. This inherent capability to integrate imaging and PDT treatment, which makes PS imaging particularly conducive to online monitoring of therapy response, was not used in the studies referenced above. It is also worth noting that the application of PDT to the treatment of OvCa has shown promise in clinical trials (Sindelar *et al*, 1991; Hendren *et al*, 2001; Hahn *et al*, 2006), although further studies are warranted to establish optimal conditions for light and PS delivery in the clinic.

The goal of this study was to explore the potential of our fluorescence microendoscope to detect disseminated small volume OvCa tumour nodules and provide more accurate and timely treatment response information than current technologies. We present characterisation of our system *in vitro*, using an OvCa 3D model system (I Rizvi^*^, JP Celli^*^, CL Evans, A Abu-Yousif, T Hasan. Adherent ovarian carcinoma cells migrate and assemble *in vitro* into heterogeneous 3D micronodules with differential treatment response. Manuscript in preparation, to be submitted. ^*^Equal contribution), and *in vivo,* using an established mouse model of disseminated OvCa (Molpus *et al*, 1996a), both of which were previously developed in our laboratory. To further demonstrate the capability of our system to monitor treatment response, we conducted endoscopic assessment before and after a single PDT treatment. In this study, PDT serves as a therapy with a variable level of cytotoxicity to provide a suboptimal dosage conducive for monitoring therapeutic efficacy.

As proof of principle, this study illustrates the significant translational potential of fluorescence microendoscopy for detection of OvCa. Specifically, this method could provide the clinician with the capability to obtain more timely and accurate treatment response feedback, which could be used to optimise treatment parameters to better predict, and hence mitigate, adverse treatment outcomes. Moreover, the capability to detect sub-millimetre tumour nodules suggests the potential utility of fluorescence microendoscopy in early detection of OvCa, if combined with serum-based or other screening tests to identify candidates for endoscopic examination.

## Materials and methods

### Fluorescence microendoscope

**Figure 1:**
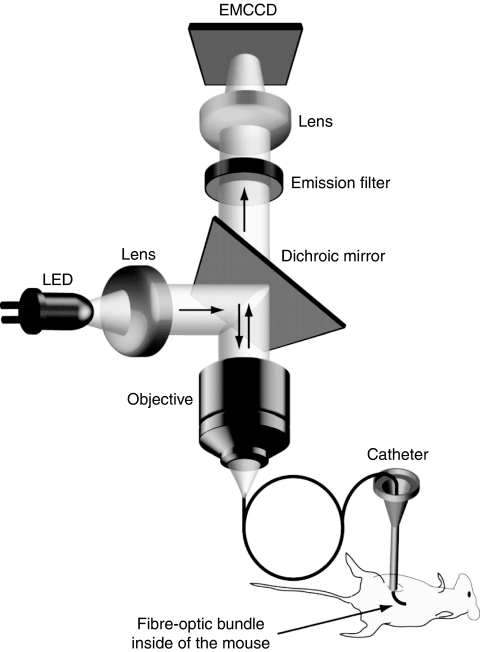
Schematic of the fibre-optic fluorescence microendoscope imaging system.

For this study, a fluorescence microendoscope with the following features was required: a miniature and flexible probe providing minimally invasive access and easy manoeuvering in the mouse intraperitoneal cavity, and imaging components suited for fluorescence imaging of BPD-MA, namely, a light source to excite BPD-MA efficiently, and a highly sensitive camera capable of real-time imaging of weak fluorescence emission in the 690–700 nm spectral window (Aveline *et al*, 1994). Although previous studies have successfully implemented commercially available endomicroscopes (Laemmel *et al*, 2004; Becker *et al*, 2007; Leung *et al*, 2009), none of these systems were well suited for BPD-MA imaging, lacking either excitation at an appropriate wavelength, sufficient sensitivity for detection of weak fluorescence emission at 700 nm, and/or having too large a probe for imaging inside a mouse. For these reasons, we instead built an instrument specifically designed for this study (Figure 1), similar to custom instruments used by others in gastrointestinal endoscopy applications (Dubaj *et al*, 2002; Muldoon *et al*, 2007, 2008), and offering the additional advantage of ease of adaptation for use with other imaging agents in future studies. The excitation source was a light-emitting diode (LXHL-LR5C, Luxeon Star LEDs, Brantford, ON, Canada) with an emission peak at 455 nm, well suited to excite the Soret band of BPD-MA without the use of an additional filter. The LED light was collimated and directed into an infinity-corrected × 10 objective (NT46-144, Edmund Optics, Barrington, NJ, USA) through a dichroic mirror (51008bs, Chroma Technology Corp., Rockingham, VT, USA). The beam was focused onto the proximal end of a 1.5 m-long, 0.8 mm in diameter, 0.35 NA flexible fibre probe (IGN-08/30, Sumitomo Electric USA Inc., Torrance, CA, USA) and transmitted to the distal end, delivering an optical power of 35 *μ*W during contact mode imaging of the sample. The coherent fibre bundle consists of a total of 30 000 cores. The core-to-core spacing, which, for contact mode imaging, dictates the optical resolution, is about 4.4 *μ*m, sufficient for detection of the presence of microscopic disease at the single cell level as required for this study. After sample excitation, fluorescence emission was collected by the fibre probe and sent through the objective, the dichroic mirror, and an emission filter (D700/40, Chroma Technology Corp.). A tube lens mounted below the camera forms the image from the objective while preserving approximately × 10 magnification of the fibre probe face on the sensor of the electron-multiplying CCD camera (Cascade 512B EMCCD, Photometrics, Tucson, AZ, USA). The Cascade 512B EMCCD was selected for this study for its high sensitivity in the spectral region of the BPD fluorescence emission peak. With a 90% sensitivity at 690–700 nm, this provides a significant advantage over a scanning-based system, by relying on a photomultiplier tube that, even with extended red performance, provides a quantum efficiency of only ∼15% in this spectral region. The EM gain and exposure time were fixed at 3000 V and 30 ms, respectively, throughout the experiments. All images were obtained using 2 × 2 binning of pixels to increase the signal-to-noise ratio for quantitative measurements and to allow for higher speed image acquisition to reduce blurring artefacts because of motion of the mouse and the hand-held fibre probe.

### Photosensitiser

A liposomal formulation of BPD-MA, also known as verteporfin (Visudyne, QLT Inc., Vancouver, BC, Canada), was used for this study. BPD-MA has been approved for use in the clinic by the Food and Drug Administration (FDA) and is under clinical trial for use with ovarian cancer. All work involving BPD-MA was conducted under conditions of minimal room lighting.

### OvCa cell cultures

The established NIH, OVCAR5 cell line, was obtained from the Fox Chase Cancer Institute (Philadelphia, PA, USA) (Hamilton *et al*, 1984). Cells were grown in RPMI-1640 media supplemented with 10% heat-inactivated FCS, 100 U ml^−1^ penicillin, and 100 *μ*g ml^−1^ streptomycin and maintained in an incubator at 37°C in 5% CO_2_. Building on 3D breast cancer models introduced by Bissel *et al* (Petersen *et al*, 1992; Muthuswamy *et al*, 2001; Debnath *et al*, 2003), we recently developed a 3D *in vitro* model of micrometastatic OvCa (I Rizvi^*^, JP Celli^*^, CL Evans, A Abu-Yousif, T Hasan. Adherent ovarian carcinoma cells migrate and assemble *in vitro* into heterogeneous 3D micronodules with differential treatment response. Manuscript in preparation, to be submitted. ^*^Equal contribution). Briefly, OVCAR5 cells were seeded as a single-cell suspension on a bed of growth factor reduced matrigel and spontaneously formed 3D acinar structures resembling micronodular ovarian cancer. The cells were grown in an assay medium containing OVCAR5 cell culture media, 5 ng ml^−1^ epidermal growth factor, and 2% Matrigel, and formed tumour nodules up to hundreds of microns in size, which served as an *in vitro* model of OvCa metastases.

### Fluorescence imaging of cell cultures

OVCAR5 cells in monolayer and 3D culture were incubated in 1.0 *μ*M BPD-MA diluted in the appropriate culture media described above to prepare for fluorescence imaging. For the time course experiment, 3D culture dishes were removed from incubation at time *t* (*t*=1, 4, 20, and 24 h) and imaged with the fluorescence microendoscope. Thereafter, 2 ml of dispase (354235, BD Biosciences, Bedford, MA, USA) was added to each dish to recover cells from Matrigel and to form a single-cell suspension. After 2 h, the cell solution was collected, along with 2 ml of media used to rinse the dish and collect any remaining cells, as well as to stop the dispase-catalysed reaction. The numbers of cells in each solution was counted using a haemocytometer. Cell suspension of 100 *μ*l was added to a well in a 96-multi-well plate. Fluorescence measurements were then obtained using a plate reader at 436 nm excitation and 690 nm emission. To convert the fluorescence measurement obtained from the samples into a concentration of BPD-MA, a calibration curve was created using cell suspensions mixed with known concentrations of BPD-MA. For each of the four time points studied, data were generated from two independently plated 3D cultures.

### Animal model

We used an OvCa mouse model previously developed in our laboratory to mimic human disseminated ovarian carcinoma (Molpus *et al*, 1996b). Female Swiss nude mice (Cox, Cambridge, MA, USA), 6–8-weeks old, were injected intraperitoneally (i.p.) with cultured OVCAR5 cells at an initial inoculum of 3.15 × 10^7^ cells in single-cell suspension in PBS. They had continual access to food and water while being housed in laminar flow racks under specific pathogen-free conditions. All animal procedures, including killing by CO_2_ inhalation, were conducted in accordance with institutional guidelines for research animal care.

### *In vivo* imaging of tumours

We performed fluorescence microendoscopy in the OvCa mice at 3–4 weeks after tumour inoculation. Each mouse was injected i.p. with 1 mg kg^−1^ body weight of BPD-MA and imaged under anaesthesia with the fibre probe introduced into the peritoneal cavity through an incision made in the abdominal wall. For comparison between microendoscope image data and pathological analysis, tissues at different anatomical sites were biopsied immediately after imaging before the mice were killed by CO_2_ inhalation. Resected tissue was fixed in 10% phosphate-buffered formalin and embedded in paraffin. Sections of 5 um thickness were stained with haematoxylin–eosin for microscopic assessment.

### *In vivo* treatment monitoring

OvCa mice were imaged at days 14 and 19 after tumour inoculation. Mice were randomly assigned to treatment and no-treatment control groups. Additional mice, which were not inoculated with tumour cells, were imaged at the same time points for healthy tissue comparison. All mice in the longitudinal monitoring study were injected with a therapeutic dose of 0.25 mg kg^−1^ body weight of BPD-MA i.p. 75 min before each imaging session, which was conducted minimally invasively by introducing the fibre probe into the i.p. cavity of the supine anaesthetised mouse through a 14-gauge catheter to traverse the abdominal wall. Sets of images of the pelvic omentum (5 images per side) and the peritoneal wall (10 images per quadrant) were obtained for each mouse at both time points while the fibre was cleaned frequently in water to remove debris from the tip. Mice that received treatment were injected with 2 ml of 0.1% intralipid solution (Intralipid Soybean Oil Emulsion; Baxter Healthcare Corporation, Deerfield, IL, USA) and each peritoneal quadrant was irradiated 90 min after BPD-MA injection with 6.25 J cm^−1^ of a 1 cm cylindrical diffusing tip fibre at 690 nm and an irradiance of 150 mW cm^−2^ (calibrated by an integrating sphere), as described earlier (Molpus *et al*, 1996a), for a total light dose of 25 J cm^−1^. Further details of the i.p. PDT treatment procedure are illustrated in Supplementary Figure 1. After the first imaging session (day 14) and treatment (when applicable), mice were allowed to wake up and resume their normal diet and activity. On completion of the second imaging session (day 19), mice were killed by CO_2_ inhalation so that tissues could be retained for pathological analysis as described above.

### Data analysis

Fluorescence images were obtained and processed by IPLab (BD Biosciences – Bioimaging Division, Rockville, MD, USA) and Matlab (The MathWorks Inc., Natick, MA, USA). To eliminate background because of the bias level set by the camera manufacturer and the leakage of reflected excitation light through the emission filter, we subtracted from each image the background image measured from water with zero fluorescence. We also compensated for the non-uniform spatial response of the system by normalising the background-subtracted images with an image from a uniform solution of 1 *μ*M BPD-MA. Both water and 1 *μ*M BPD-MA solutions were freshly prepared and imaged at the beginning of each imaging session. Pixel values within each image were calibrated as follows: 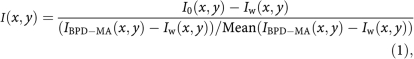 where *I*_0_, *I*_w_, and *I*_BPD-MA_ are the raw images of a sample, water, and 1 *μ*M BPD-MA solution, respectively. Mean represents the mean value of all pixels (*N*) within the endoscope's field of view (0.8 mm in diameter). This normalisation term minimises sensitivity variation because of occasional system realignment.

The fluorescence intensity of BPD-MA in the 3D cultures of tumour nodules was calculated as follows. First, individual tumour nodules were identified by segmentation on the basis of selection of pixels with intensity values above a threshold that corresponded to background fluorescence from the culture media containing BPD-MA. Segmentation was based on the triangle algorithm, which was readily available as a built-in utility of the IPLab software package. In this technique, a line connecting the minimum and maximum values in the histogram of intensities of all pixels in an image is established. The distances between the line and all intensity values are computed. The intensity value that has the shortest distance to the line is set as the threshold value for separating objects from the background. The fluorescence intensity of each segmented nodule was calculated by spatial averaging over all interior pixels. The same segmentation approach was used to outline tumour nodules of OvCa mice for the purpose of size determination *in vivo*.

For the treatment monitoring study, in which large sets of images were processed before and after treatment, mean BPD-MA fluorescence values were calculated by means of custom scripts to batch-process entire directories of data. For each image, mean fluorescence intensity was determined by averaging all pixels within the circular field of view of the endoscope. The field of view was automatically determined in each image (to account for slight displacements of the field of view with respect to the CCD chip) using a custom routine that uses a combination of a wavelet transform-based edge detection and circle detection algorithms. This mean value was corrected for the background level as described above and normalised to 1 μM BPD-MA control solution. Normalised values were grouped by site (pelvic omentum or peritoneal wall) for each mouse at each time point to calculate the overall mean for each site and the percentage change in fluorescence intensity at that site from day 14 to 19 as described further below.

## Results

### Validation of fluorescence imaging in cell cultures

**Figure 2:**
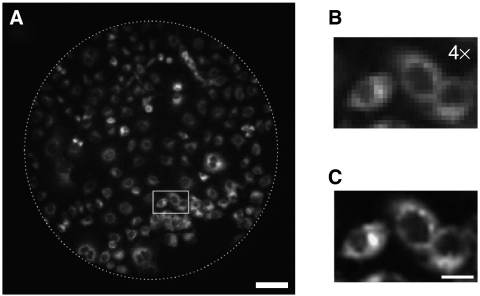
Fluorescence image of monolayer OVCAR5 cells *in vitro* incubated in BPD-MA. (**A**) Endoscope image. Dotted line indicates the field of view of 0.8 mm. Scale bar is 100 *μ*m. (**B**) Enlarged image of the area marked in (**A**) (square). (**C**) Same as B after removing pixelation and enhancing contrast by software. The image shows localisation of BPD in the cytoplasm. Scale bar is 25 *μ*m.

To establish the capability of our fluorescence microendoscope to resolve single cells, we first imaged OVCAR5 cells grown in monolayer and incubated with BPD-MA for 4 h (Figure 2). Our instrument was able to resolve individual cells. The ability to observe some degree of subcellular structure is consistent with our estimate of a 4.4 *μ*m optical resolution based on the spacing of fibre cores noted above. Within individual cells, the diffuse pattern of BPD-MA fluorescence localised to the cytoplasm is characteristic of the intracellular distribution of BPD-MA observed in previous studies (Figure 2B and C) (Osaki *et al*, 2006).

**Figure 3:**
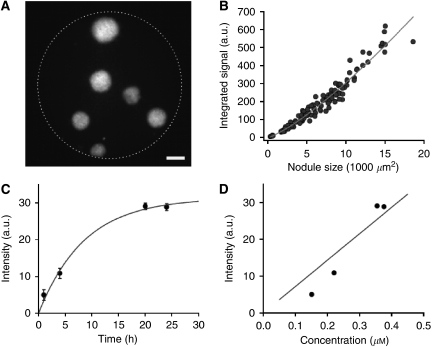
Validation of fluorescence imaging in 3D cultures. (**A**) Representative fluorescence image of 3D culture of tumour nodules incubated in BPD-MA. Scale bar is 100 *μ*m. (**B**) Fluorescence intensity of BPD-MA in tumour nodules plotted against nodule size. The plotted data are fit to a power law (line) with *k*=1.27, with *R*^*2*^=0.94. (**C**) Uptake of BPD-MA by 3D cultures over time measured with the fluorescence microendoscope. The error bars represent standard deviations. The trend line is a single exponential fit. (**D**) Uptake of BPD-MA by 3D cultures over time measured with the fluorescence microendoscope *vs* plate reader measurements from cells of the same disaggregated tumour nodules. The linear regression, represented by the solid line, suggests the consistency between these two methods.

To establish our instrument's capability for quantitative measurements, BPD-MA fluorescence intensity from *in vitro* 3D tumour cultures was correlated with nodule size and fluorescence from disaggregated 3D nodules. Figure 3A is a representative image of 3D cultures of tumour nodules imaged 24 h after incubation in BPD-MA. As anticipated, the integrated BPD-MA fluorescence signal fluorescence from tumour nodules was found to increase with nodule size (Figure 3B). A power law curve fit indicated a relationship given by *I α a*^*k*^, where *a* is the nodule area (in our 2D image) and *k*=*1.27*. This power law scaling is within reason for the characteristics of our model and light collection. We would expect *k*=1 for clusters of monolayer cells in which case light would be collected from an essentially flat plane with intensity scaling linearly with the area (determined by the number of cells in the cluster). For the ideal scenario of total light collection from the full volume of perfectly spherical clusters of cells, we would expect intensity to scale with area to the 3/2 power (*k*=1.5), corresponding to a linear relationship with volume. The fact that the measured power law dependence of *k*=1.27 lies in between these two limiting cases is not surprising, in view of the non-3D light collection from the finite depth of focus of the imaging device, combined with the fact that the *in vitro* micronodules are in fact not perfectly spherical.

As illustrated in Figure 3C, during the 24 h BPD-MA incubation, there was a time-dependent increase in fluorescence intensity. The measured data fitted well with an exponential model given as *I*=*I*_0_^*^(1−e^−k*t^), where *I*_0_=3.1 × 10^4^ and *k*=0.12 (Figure 3C). To validate that the fluorescence intensities measured by the microendoscope were reflective of the actual BPD-MA concentrations in the 3D acini, we disaggregated the *in vitro* nodules and measured fluorescence from dissociated cells with a plate reader and determined concentration by a standard curve. Figure 3D illustrates the strong correlation between fluorescence intensities as measured by the microendoscope and the plate reader. A linear fit of these two independent measurements gave a Pearson's coefficient of regression *R*^*2*^=0.84.

### *In vivo* imaging of micrometastases

**Figure 4:**
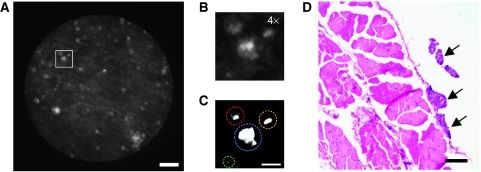
*In vivo* detection of micrometastases. (**A**) Fluorescence image of small tumour nodules (tens of microns in size) detected in OvCa mouse by the fluorescence microendoscope using BPD-MA as the contrast agent. (**B**) Enlarged image of the area marked in (**A**) (square). (**C**) Contrast-enhanced image of (**B**). Four high-intensity spots (circles) are observed. (**D**) H & E stain of a 5 *μ*m-thick section from the same region. Arrows indicate tumour nodules. Scale bars are 100 *μ*m in (**A**) and (**D**), and 25 *μ*m in (**C**).

Microscopic tumour nodules were identified during imaging sessions by slowly scanning the tip of the fibre probe over peritoneal surfaces inside anaesthetised mice and acquiring images with particular emphasis on the pelvic and subgastric omenta, the peritoneal wall, and the bowel mesentery, known to have high rates of tumour occurrence. To determine the size of the microscopic tumour nodules detected, images obtained in the mouse i.p. cavity were compared with the pathological analysis of tissues resected from the same sites. Figure 4 shows a fluorescence image (A–C) and H & E stain (D) of a section obtained from the same tissue. This result indicates that fluorescence endoscopy can detect tumour nodules as small as a few tens of microns (arrows).

We compared the fluorescence images of 171 samples biopsied from eight OvCa mice with the results of pathological analysis. Microendoscopic images and correspsonding histopathology specimens were assessed by an investigator to identify samples that were positive for tumour, as identified by the presence of any visible nodules. Of the 141 samples determined to be neoplastic by pathological analysis, 121 showed evidence of tumour nodules by fluorescence microendoscopy, with each tumour-positive field/specimen being weighted equally, regardless of the number of nodules. Of the 30 samples determined to be normal by pathological analysis, 16 seemed to be tumour free by fluorescence microendoscopy. Hence, with BPD-MA as the contrast agent, the sensitivity and specificity of fluorescence microendoscopy for detecting tumour nodules were 86% and 53%, respectively.

### Monitoring of PDT treatment response

**Figure 5:**
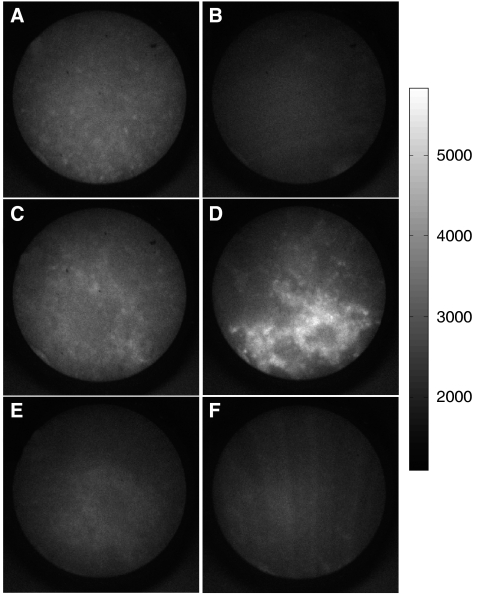
Representative longitudinal monitoring microendoscopic images from the peritoneal wall of treated, untreated, and normal mice at days 14 and 19 shown with an intensity scale for comparison. (**A**, **B**) Images from a PDT-treated mouse at days 14 and 19, respectively. (**C**, **D**) Images from an untreated mouse at days 14 and 19. (**E**, **F**) Images from a normal (non-tumour bearing mouse) at days 14 and 19. In general, the intensity of BPD-MA fluorescence was observed to decrease from day 14 to 19 in mice that received PDT treatment, whereas the opposite trend was evident in untreated mice. Microendoscopic images obtained from normal mice showed low levels of fluorescence intensity independent of the imaging time point.

**Figure 6:**
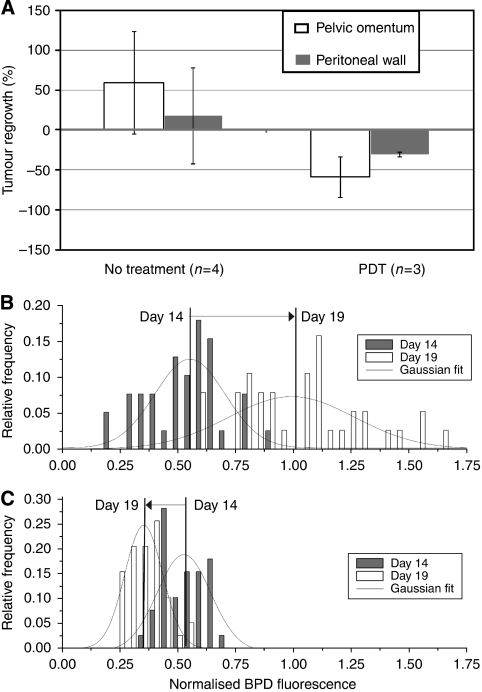
Treatment-monitoring data. (**A**) Mean percentage tumour regrowth for all mice in the treatment and control groups obtained from sets of images at days 14 and 19 for pelvic omentum (white) and peritoneal wall (grey). Error bars represent mouse-to-mouse variation. (**B**) Distribution of fluorescence intensities from day 14 images (grey bars) to day 19 images (white bars) for a given mouse in the no-treatment group, showing longitudinal shift to the right (increasing fluorescence intensity). Histograms were generated by grouping normalised intensity values into bins of width 0.05 and are plotted as relative frequency of occurrence *vs* intensity (bin end). (**C**) Distribution of fluorescence intensities from images of the peritoneal wall for a given mouse in the PDT treatment group showing a downwards shift in fluorescence intensity as compared with the no-treatment control. Distributions in (**B**) and (**C**) are obtained from 40 measurements per time point, per mouse.

Having established the capability of our fluorescence microendoscope for sensitive detection of OvCa, we conducted a pilot study to evaluate the potential of our technique to serve as a quantitative reporter of therapeutic response. OvCa mice were imaged immediately before PDT treatment, or control injection of PS, at 14 days after tumour inoculation and reassessed at day 19 (at which point only the day 19 reinjection is relevant to the measured PS fluorescence signal because of the rapid clearance rate of BPD-MA from peritoneal tissues, which is reported in Supplementary Figure 2). Representative images obtained at each time point from mice in each treatment group and from non-tumoured control mice are shown in Figure 5. To quantitatively evaluate the observed changes in treated and untreated mice, it was necessary to develop a robust method for minimally invasively surveying disseminated OvCa, in which tumour nodules are spread in an inhomogeneous manner over a large surface area in the i.p. cavity. To achieve this while minimising sampling bias, we took the approach of acquiring large sets of image data and developed a batch-processing technique to generate distributions of BPD-MA fluorescence intensities by region. For each mouse, at each time point, we obtained sets of images of 40 sites on the visceral peritoneum, divided equally over 4 quadrants, and 10 sites on the pelvic omentum, over both sides of the pelvis. We then tracked changes in BPD-MA fluorescence intensity as a reporter of tumour nodule volume, building upon the correlation established above from our 3D tumour model (Figure 3). Normalised fluorescence intensities from each image determined by batch-processing directories of image data were grouped and further processed to calculate the average measured percentage tumour regrowth (or destruction as indicated by a negative percentage) by site for untreated and PDT-treated mice (Figure 6). This approach, although not taking full advantage of the 2D image data provided by the endoscope, provided a robust means of quantifying disease progression while avoiding potential pitfalls of extensive automated manipulation of large sets of image data to count and measure individual nodules in sets of images. The latter could be especially problematic in cases in which the disease becomes caked to completely fill the microendoscope field of view (particularly in the omentum tissue), in particular while imaging with a lower-contrast therapeutic dose of BPD-MA.

Our microendoscopy imaging and analysis reports that disease developed unchecked in untreated mice, with positive mean percent changes from day 14 to 19 of +59.3% and +17.7% for the pelvic omentum and peritoneal wall, respectively (Figure 6A). In treated mice, this trend is reversed with negative values of regrowth (indicating mean tumour destruction) of −58.8% and −30.2% in the same sites, consistent with the modest, single dose of PDT applied here. Comparison of treatment and control groups on the basis of images from the pelvic omentum was clearly significant with a *P*-value of 0.027 (two-tailed Student's *t*-test), in contrast to the comparison based on peritoneal wall images with a *P*-value of 0.21. The larger separation in response between treated and untreated groups on the basis of images of the pelvic omentum is likely because of a higher rate of proliferation for cells implanted on these energy-rich fat pads relative to the peritoneal wall, leading to more significant growth in the untreated group during the time between imaging sessions. Conversely, in treated mice, the lipophilicity of verteporfin may have led to larger local accumulation of BPD-MA in the microenvironment of those tumour nodules that are surrounded by fatty tissues, thus leading to a higher local cytotoxic effect in the pelvic omentum and further enhancing significance in measured treatment response. In addition, some level of variability in tumour aggressiveness and response to treatment from mouse to mouse is typical of this model of disseminated OvCa (Molpus *et al*, 1996a, 1996b). In the images obtained from tumour-free mice, there was no significant change in fluorescence intensity between imaging sessions for either of the sites probed.

The bottom two panels of Figure 6 detail the calculation of the change in BPD-MA fluorescence between the two imaging time points from a given untreated mouse (Figure 6B) and PDT-treated mouse (Figure 6C) used in the calculation of group averages. These histograms were prepared by grouping intensity values from individual images into bins of width 0.05 and plotted as relative frequency of files occurring in a particular bin *vs* normalised intensity. The image-to-image fluctuations in intensity at each time point reflect the spatial variation in nodular studding and occasional regions of more caked disease encountered by the fibre probe as it scanned over peritoneal surfaces. The Gaussian fits to the distributions of fluorescence intensities at each time point indicate the calculation of the mean value that increases from day 14 to 19 for the untreated mouse (indicating uninterrupted tumour growth) in Figure 6B. In contrast, the shift of the distribution of fluorescence intensities towards lower values from day 14 to 19 for a mouse that received PDT treatment in Figure 6C shows some tumour destruction for a PDT dose of 25 J cm^−1^.

## Discussion

In this study, we show the ability of a custom fluorescence microendoscope to detect OvCa micrometastases on the order of tens of microns in diameter (Figure 4) – a feature that has not been shown by conventional imaging modalities such as ultrasound, MRI, or PET. We note that this size is an order of magnitude smaller than those found in previous reports describing PS fluorescence imaging in OvCa animal models or patients (Chan *et al*, 2002). In the context of treatment assessment, the ability to detect such small nodules could be invaluable to clinicians by providing feedback early in the treatment cycle rather than having to wait until nodules have become large enough for detection by laparoscopy or surgical reassessment, at which point the disease has advanced to a stage with poor prognosis. The microendoscope's high sensitivity (86%) to tumour nodules, which translates to a false-negative rate of 14% as compared with 31% reported for white light imaging in a similar murine model (Sheth *et al*, 2009), may hold the key to a more effective detection of OvCa.

Our choice of BPD-MA, a potent photosensitiser, as a fluorescence contrast agent made PDT treatment a useful therapeutic tool to assess the treatment monitoring capability of the instrument, enabling us to treat and monitor response using the same molecule. Using BPD-MA in this dual role as a therapeutic and sensitive tumour recognition agent, we show the potential of our technique to serve as a quantitative tool to evaluate acute response to treatment using fluorescence as a reporter of tumour volume. We observed evidence of tumour destruction in mice that received PDT treatment, whereas untreated mice showed uninterrupted progression of disease from microendoscopy of the pelvic omentum, as well as the peritioneal wall. The challenges of sampling the large surface area of the latter site suggest the benefits of incorporating this technology with traditional white light laparoscopy, which is already in clinical use. In fact, because of the flexible imaging fibre used in the microendoscope, the two methods could be highly complementary. One could imagine a clinician seamlessly going back and forth between the laparoscope and the microendoscope, with the wide field imaging of the former providing a cursory identification of suspicious regions to be further interrogated with higher sensitivity and microscopic details using the microendoscope. The diagnostic efficiency of fluorescence microendoscopy, which, as with any epi-fluorescence-based detection strategy, is limited in its ability to penetrate below the tissue surface, could be further enhanced by integration with a complementary imaging modality to provide depth-resolved structural information.

Our results specifically indicate the promise of fluorescence microendoscopy to fill the vitally needed niche of a minimally invasive technique for treatment monitoring. To follow-up on these findings, further studies are warranted to extend these findings from the acute treatment response examined here to assess the capability of this technique to predict long-term outcomes to more extensive therapeutic regimens in survival studies. It should be further noted that, although our use of a PS made PDT a convenient modality for testing this technology, this instrumentation is not specific to monitoring PDT treatment and could be used in the same capacity as other modalities, including surgery and chemotherapy.

Future improvements will include the incorporation of targeted imaging contrast agents to improve specificity for imaging tumours. However, it is encouraging to note that even while implementing our fluorescence microendoscope without a targeted imaging agent, the tumour-to-normal ratios of BPD-MA in fluorescence images were found to be between 2 and 3, with an overall sensitivity of 86% for tumour detection. We anticipate that with the development of more specific tumour-targeted imaging contrast agents, a substantial improvement in both tumour-to-normal contrast and specificity will be realised. This is part of an ongoing effort in our laboratory to develop suitable constructs to explore the use of nanoparticle formulations to allow a high degree of customisation in combining fluorescence imaging agents with appropriate binding sites for specific small molecule targets. Furthermore, one could envision designing theranostic-targeted nano-constructs that could carry not only fluorophores but also therapeutic agents. Such ‘smart’ constructs could be used in conjunction with fluorescence microendoscopy for the diagnostic therapy of cancer (Sumer and Gao, 2008).
