# Supplementary material for: In vivo high-resolution fluorescence microendoscopy for ovarian cancer detection and treatment monitoring
Source: Br J Cancer. 2009 Nov 17;101(12):2015–22. doi: 10.1038/sj.bjc.6605436 (PMC2795438; doi:10.1038/sj.bjc.6605436)
Supplement: Supplementary Information [file 6605436x1.doc]

**Supplemental Material**

**
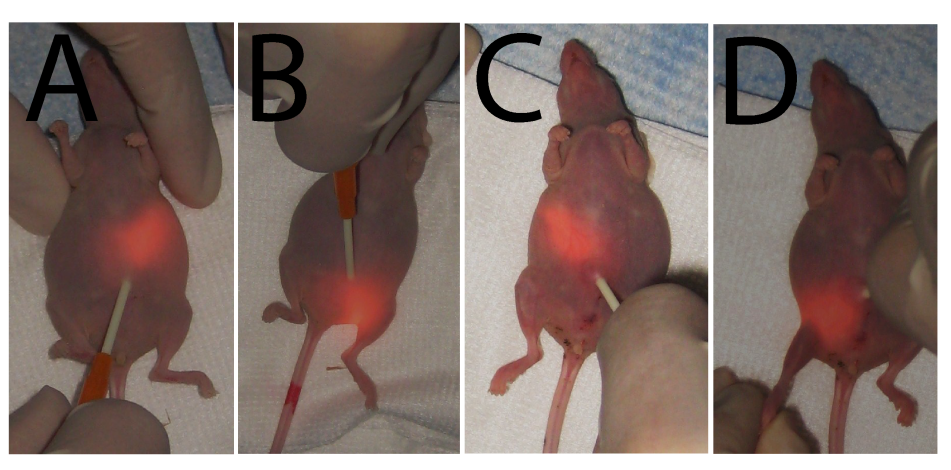
**

**Supplemental Figure 1.** Intraperitoneal PDT in a murine model of disseminated OvCa. Irradiation of the upper left (**A**), lower left (**B**), upper right (**C**), and lower right (**D**) peritoneal quadrants with 690 nm light is shown. To each quadrant an equal light dose of 6.25 J/cm of a 1 cm cylindrical diffusing tip fiber (calibrated via integrating sphere to an irradiance of 150 mW/cm^2^) was delivered in the presence of a 0.1% intralipid solution to improve the uniformity of light scattering. The diffusing tip fiber was inserted into the peritoneal cavity via 14-gauge catheter. In each panel the approximate region being irradiated is evident from the diffuse red light transmitted through the skin from the fiber tip inside the mouse.

**
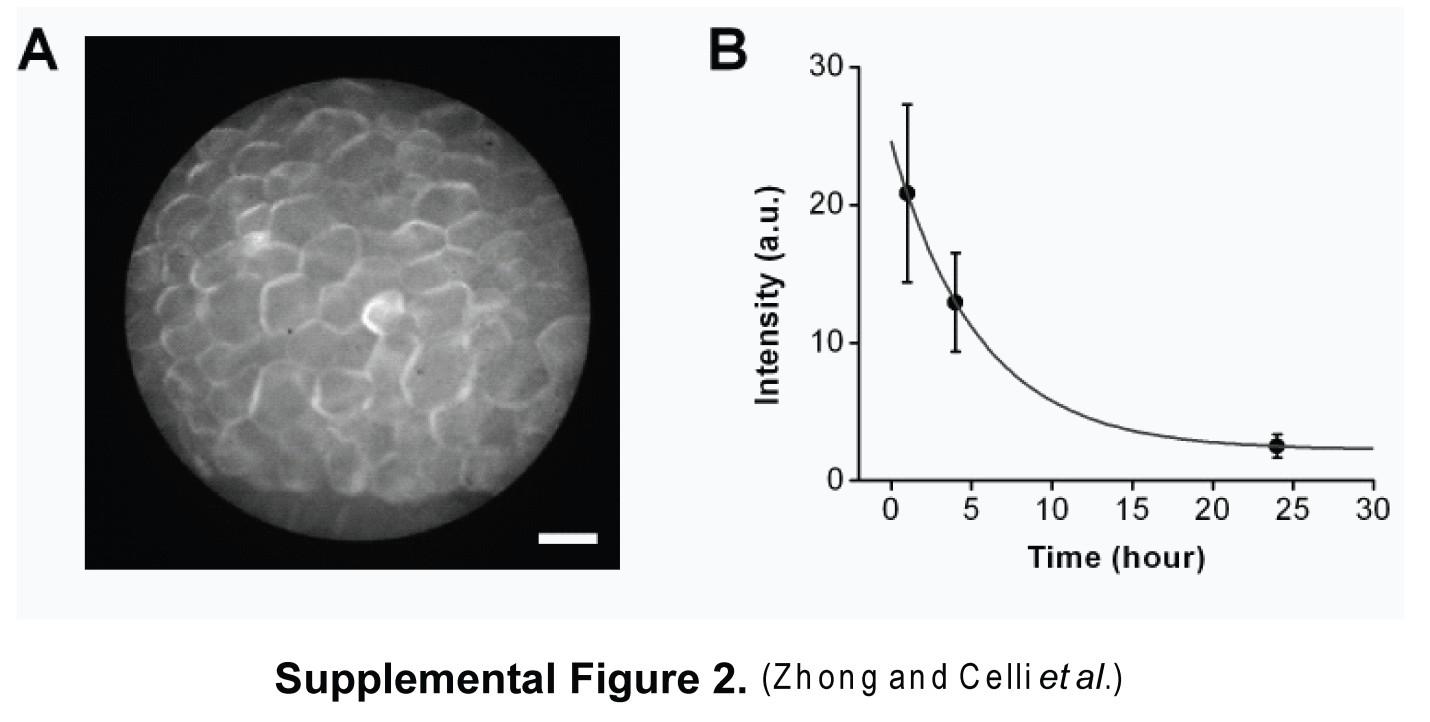
**

**Supplemental Figure 2.** *In vivo* time-dependent BPD-MA fluorescence intensity measured by microendoscopy. (**A**) Fluorescence image of pelvic omentum of a mouse injected with BPD-MA. Scale bar is 100 μm. (**B**) Fluorescence intensity from the pelvic omentum of mice measured at different time points after BPD-MA injection, demonstrating nearly complete clearance by 24 hours. The error bars represent the variance in measurements from 3 mice per time point.
